# Multiple focal pulvinar projection fields in the macaque cortex

**DOI:** 10.1162/imag_a_00202

**Published:** 2024-06-26

**Authors:** Mathilda Froesel, Simon Clavagnier, Quentin Goudard, Qi Zhu, Wim Vanduffel, Suliann Ben Hamed

**Affiliations:** Institut des Sciences Cognitives Marc Jeannerod, UMR5229, CNRS-University of Lyon 1, Lyon, France; Department of Neurosciences, Laboratory of Neuro- and Psychophysiology, KU Leuven Medical School, Leuven, Belgium; Cognitive Neuroimaging Unit, INSERM, CEA, Université Paris-Saclay, NeuroSpin Center, Gif/Yvette, France; Athinoula A. Martinos Center for Biomedical Imaging, Massachusetts General Hospital, Charlestown, MA, United States; Department of Radiology, Harvard Medical School, Boston, MA, United States

**Keywords:** pulvinar, macaque, resting-state, fMRI

## Abstract

The pulvinar, the largest nucleus of the thalamus, is functionally heterogeneous and involved in multiple cognitive functions. It has been proposed to act as a functional hub of cortical processes due to its extensive reciprocal connectivity with the cortex. However, its role in cognition is not fully understood yet. Here, we posit that an improved understanding of its functional connectivity with the cortex is needed to better capture the cognitive functions of this nucleus. To address this question, we characterize the pulvino-cortical functional connectivity along the ventro-dorsal, antero-posterior, and medio-lateral axes, using awake resting-state data from 10 adult macaques. We first report two global cortical functional connectivity gradients along the antero-posterior and ventro-dorsal pulvinar gradients that match remarkably well the structural connectivity gradients described by anatomical approaches. In addition to these global gradients, multiple local cortical pulvinar projection fields can be identified at the sulci level such as in the lateral sulcus (LS), the intraparietal sulcus (IPS), the principal sulci (PS), and the anterior cingulate cortex (ACC). For most sulci, we show that functional pulvino-cortical projection fields follow the major anatomical axis of these different sulci (e.g., the ventro-dorsal axis for the LS and the antero-posterior axis for the IPS). Other sulci, such as the superior temporal sulcus, the posterior cingulate cortex, or the central sulcus, display multiple projection fields from the pulvinar. Although substantial inter-individual differences exist, the general functional connectivity patterns are remarkably consistent across hemispheres and individuals. Overall, we propose that these multiple pulvinar projection fields correspond to a fundamental principle of pulvino-cortical connectivity and that a better understanding of this connectional organization will shed light on the function of pulvino-cortical interactions and the role of the pulvinar in cognition at large.

## 1 Introduction

The pulvinar, the largest and most posterior nucleus of the thalamus, is characterized by widespread anatomical connections with the rest of the brain. In primates, based on the highly complex cortico-pulvino-cortical connectivity and the pulvinar’s synaptic organization with typical feedback and feedforward projections, the pulvinar has been proposed to act as a functional hub or modulator of cortical processes (Benarroch, 2015;Rouiller & Welker, 2000;Saalmann & Kastner, 2011;Sherman, 2007;Sherman & Guillery, 2006;Shipp, 2003). The anatomy of this higher-order subcortical nucleus is also very heterogeneous. Primate pulvinar is classically divided in several sub regions based on their specific cytoarchitectonic and chemoarchitectonic properties, namely the anterior, medial, lateral, and inferior subdivisions of the pulvinar, each containing even smaller subdivisions (Gutierrez et al., 1995,^2000^;Olszewski et al., 1952;Stepniewska & Kaas, 1997;Walker, 1938). For example, the medial pulvinar is divided in medio-lateral and medio-medial nuclei; the lateral pulvinar is divided into a ventro-lateral subregion and a dorso-lateral part traditionally called dorso-medial lateral pulvinar (PLdm). The inferior pulvinar is divided into the posterior, medial, and central inferior pulvinar. In humans, functional pulvinar parcellations have mostly relied on meta-analyses of task-related neuroimaging studies and resting-state-based clustering analyses (Barron et al., 2015;Guedj & Vuilleumier, 2020). Both studies describe five pulvinar functional clusters, although they do not locate them at the exact same anatomical position. The meta-analysis defines an inferior, lateral, medial, anterior, and superior pulvinar cluster (Barron et al., 2015) while the resting-state analysis splits the pulvinar into a dorso-medial, ventro-medial, lateral, anterior, and inferior cluster (Guedj & Vuilleumier, 2020). These results demonstrate that, functionally and anatomically, the subregions of the pulvinar are complex to define.

In the present study, we analyzed functional pulvino-cortical connectivity at the spatial resolution of individual fMRI voxels along the ventro-dorsal, antero-posterior, and medio-lateral axis. Based on the literature, we predict that the pulvinar follows a functional connectivity gradient with the cortex that matches the anatomical pulvino-cortical connectivity organization, namely a ventro-dorsal pulvinar gradient that maps onto an antero-posterior cortical functional gradient (Froesel et al., 2021). We also predict locally-specific topographically organized projections from the pulvinar to the cortex. Such local pulvinar projection patterns have been reported in specific cortical regions such as the superior temporal sulcus or MT (Grimaldi et al., 2016;Mundinano et al., 2019). To investigate both global and local functional connectivity patterns between the pulvinar and the cortex, we performed resting-state fMRI analyses from 10 awake fixating monkeys using single voxels as seeds for whole-brain functional connectivity analyses. We report that, globally, as predicted by the literature, the pulvino-cortical connectivity mainly follows a ventro-dorsal and antero-posterior gradient. Most importantly, we additionally show multiple focal functional pulvinar projection fields at the cortical level, mostly organized around the main sulci. In other words, the entire pulvinar projects multiple times on the cortex, thus creating multiple pulvinar projection fields in the cortex. Such focal functional pulvinar projection fields were found in the lateral sulcus, the superior temporal sulcus, the intraparietal sulcus, the prefrontal cortex, the orbito-frontal cortex, the central sulcus, and the anterior cingulate cortex. Although there are substantial inter-individual differences, these focal projection fields are consistently observed across hemispheres and multiple subjects. This calls for a reappraisal of the organization of pulvino-cortical functional connectivity loops.

## 2 Material and Methods

### 2.1 Subjects

Ten rhesus monkeys (Macaca mulatta) participated in the study (6 females, 4 males). Animal care procedures met all Belgian and European guidelines and were approved by the KU Leuven Medical School.

### 2.2 Experimental setup

During the scanning sessions, monkeys sat in a sphinx position in a plastic monkey chair (Vanduffel et al., 2001) facing a translucent screen. Visual stimuli were retro-projected onto this translucent screen. Eye position (X, Y, right eye) was monitored thanks to a pupil–corneal reflection tracking system (RK-726PCI Iscan) at 120 Hz. During the resting-state acquisitions, animals were required to maintain fixation into a 2 x 3 visual degrees tolerance window around a small red cross and were rewarded with apple juice for it.

### 2.3 Scanning procedures

In this study, in-vivo MRI scans were performed with a 3T MR Siemens Trio scanner and PrismaFit in Leuven, Belgium.

#### 2.3.1 Anatomical MRI acquisitions

Accompanying T1-weighted anatomical images were obtained during different sessions using a magnetization-prepared rapid gradient echo (MP-RAGE) sequence (TR = 2200 ms, TE = 4.06 ms, voxel size = 0.4 mm by 0.4 mm by 0.4 mm). During the anatomical scans, the animals were sedated using ketamine/xylazine (ketamine 10 mg/kg I.M. 1 xylazine 0.5 mg/kg I.M., maintenance dose of 0.01–0.05 mg ketamine per minute I.V.).

#### 2.3.2 Functional MRI acquisitions

Functional MRI acquisitions were as follows. Before each scanning session, a contrast agent, composed of monocrystalline iron oxide nanoparticles, Molday ION™ or Feraheme, was injected into the animal’s saphenous or femoral vein to increase the signal-to-noise ratio (Leite et al., 2002;Vanduffel et al., 2001). We acquired gradient-echo planar images covering the whole brain (40 slices, 84-by-84 in-plane matrix, flip angle = 75°, repetition time (TR): 2.00 s or 1.4 s depending on the monkey; echo time (TE): 17-19 ms; resolution: 1.25 x 1.25 x 1.25 mm voxels) with an eight-channel phased-array receive coil; and a saddle-shaped, radial transmit-only surface coil (MRI Coil Laboratory, Laboratory for Neuro- and Psychophysiology, Katholieke Universiteit Leuven, Leuven, Belgium, seeKolster et al., 2014). Specific parameters varied across monkeys (Table 1).

**Table 1.** Detailed acquisition parameters for each of the 10 monkeys.

Monkeys	Number of sessions	Number of runs	Run length (pulses)	TR (s)	Total (min)
Monkey CH	2	31;16	301	2	471.56
Monkey FE	1	8	305	2	81
Monkey FI	2	24;11	301	2	351.16
Monkey IN	1	13	300	2	130
Monkey JA	2	41;20	422	1.4	600.64
Monkey KA	2	23;9	301	2	321.06
Monkey LA	2	20;12	301	2	321.06
Monkey LE	2	21;9	300	2	300
Monkey NA	3	37;5;13	422	1.4	541.56
Monkey TH	1	9	905	2	271.5

Functional volumes were corrected for head motion within and across sessions and slice time. They were linearly detrended, coregistered on the anatomical image, and then normalized to the template space F99 with the resolution 1 mm isotropic (http://sumsdb.wustl.edu/sums/macaquemore.do). A spatial smoothing was then applied with a 2-mm FWHM Gaussian Kernel.

The data are reused from previous studies from Wim Vanduffel’s lab (Mantini et al., 2011,^2013^;Pijnenburg et al., 2019). These data were produced over different studies. The specific acquisition parameters varied across studies and animal behavior (longer runs and higher number of runs were acquired for monkeys achieving better fixation), resulting in a more robust evaluation of functional connectivity in these monkeys. The longest cumulated resting-state scanning duration (81 min), the shortest duration of individual runs (10 min), and the lowest number of runs (8 runs) combined is in the standard recommendations in the field (Birn et al., 2013). As a result, the variability induced by the differences in scanning parameters is expected to be minimal, and if anything, it is expected to enhance the robustness of our results, as our approach highlights common connectivity patterns across all monkeys, irrespective of low-level methodological differences.

### 2.4 Data analysis

Runs were analyzed using AFNI (Cox, 1996) and FSL (FSL, RID:birnlex_2067;Jenkinson et al., 2012;http://fsl.fmrib.ox.ac.uk/fsl/fslwiki/).

**Fig. 1.**
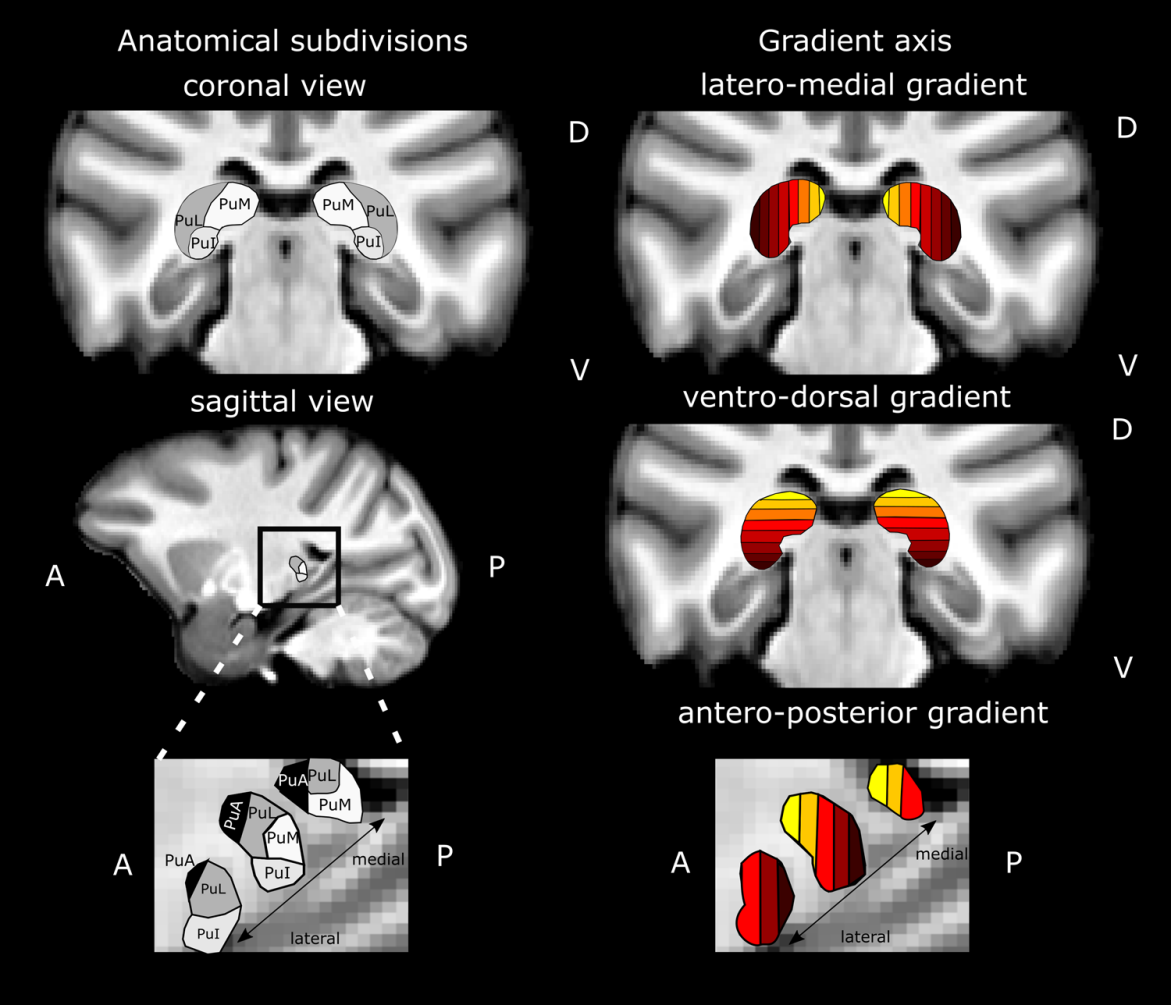
Anatomical pulvinar subdivisions and definition of latero-medial, ventro-dorsal, and antero-posterior analysis gradients. The gradients are not based on anatomical subdivisions but can partially match with them depending on the chosen analysis gradient. For example, due to the shape of the pulvinar, PuI and PuM sub-regions will share the same color code along the ventro-dorsal gradient while PuM will be associated with lighter colors than PuL, along the latero-medial and antero-posterior gradients (See SupplementaryFig. S1for quantitative evaluations of this). PuL and PuA are respectively captured by the latero-medial gradient and antero-posterior gradient. PuI: inferior pulvinar; PuL: lateral pulvinar; PuM: medial pulvinar; PuA: anterior pulvinar.

The pulvinar of each hemisphere was first delineated thanks to the NeuroMaps Atlas (Rohlfing et al., 2012) and the SARM atlas (Hartig et al., 2021) themselves coregistered on the F99 template (Fig. 1). However, due to individual variability in the 3D shape of the pulvinar (Shipp, 2001), even if their data were registered and normalized on the same template, adjustments were done by hand for some monkeys. These adjustments were minimal and aimed at optimizing the fit to the monkey’s anatomical specificities (see SupplementaryFig. S1A, Bfor atlas registration and hand adjustment and C for individual variability).

#### 2.4.1 Seed-to-whole-brain analysis

Individual functional voxels composing the pulvinar were defined as individual regions of interest. These ROIs were then used as seeds for a*seed-to-whole-brain analysis*. We performed this type of analysis for each ROI (voxel) and each run of each monkey using FSL. Head movements were used as regressors of non-interest. A Fisher’s r-to-z transformation was applied to the obtained correlation matrix. We then performed, using AFNI, a one sample t-test on the resulting maps of all the runs for each ROI. This statistical analysis resulted in a single correlation map (z-score) for each pulvinar ROI and each monkey, showing only the voxels that significantly correlated across all runs (p < 0.001).

#### 2.4.2 “Winner take all” analysis

All these correlation maps were compared to define for each voxel of the cortical map the pulvinar ROI with which the functional correlation value was highest (winner take all — within individual subjects). To do so, we took all the correlation maps of each pulvinar ROI (slice) and pooled them into a larger matrix (4D matrix: X x Y x Z x Pulvinar ROI identifier, the pulvinar ROI identifier indexing the ROI along either the latero-medial, the ventro-dorsal, and the antero-posterior axes). Using Matlab®, we then identified for each voxel of the cortex the pulvinar ROI with which it was maximally correlated. For each voxel of the cortex, we therefore created a map assigning for each voxel the ROI of the pulvinar with which it correlates most. Thus, this “winner take all” analysis resulted in a single correlation map for each monkey where each cortical voxel was associated with the pulvinar ROI it correlated the most with (or no ROI if the statistical threshold in the z-score map was not reached). Thus, this map shows with high spatial resolution, the region of the pulvinar with which each cortical voxel correlates most. This is not an exclusive map, in the sense that it does not mean that cortical voxels do not also correlate with other pulvinar ROIs. This procedure was independently performed for the pulvinar ROIs along the latero-medial, the ventro-dorsal, and the antero-posterior axes.

For visualization purposes, three color gradients have been created. The functional connectivity of pulvinar ROIs was grouped by slices defined along the latero-medial axis, the antero-posterior axis, and the ventro-dorsal axis such that their color corresponded to a specific slice in the pulvinar along the specified axis (Fig. 1). Each slice could thus include voxels from several sub-parts of the pulvinar. The proportions of voxels belonging to each pulvinar sub-part, that is, anterior, medial, lateral, and inferior pulvinar, per slice are indicated in SupplementaryFigure S2.

To observe the overall correlation across monkeys, we computed a weighted average of the individual monkey correlation maps. To do so, we summed the maps across all monkeys and then divided the value of each voxel by the number of monkeys for which functional connectivity with the pulvinar was significant. In addition, we kept only voxels for which at least half of the monkeys presented a significant correlation.

To investigate the orientation of the pulvino-cortical connectivity at the local scale, that is, at the scale of the sulcus, we attributed to each pulvinar slice or cortical location along a given sulcus a value describing its relative position along the ventral to dorsal (lower values for ventral and higher value for dorsal), the posterior to anterior, and the medial to lateral axes. We then performed linear regressions between the position within a given sulcus and the position of the most correlating pulvinar slices, across monkeys, for each hemisphere independently. This allowed us to investigate the spatial organization of the projections from the pulvinar to a given sulcus, addressing, for example, if the most dorsal slices of the pulvinar correlated most with the most dorsal slices of a given sulcus, or whether the most ventral slices of the pulvinar correlated most with the most ventral slices of this same sulcus. SupplementaryTable S1summarizes the statistical outcome of these linear regressions for all selected sulci and both hemispheres.

## 3 Results

The anatomical organization of the pulvinar in well-established sub-nuclei does not fully account for the functional organization of the pulvinar, nor for its functional connectivity with the cortex (reviewed inFroesel et al., 2021). Therefore, we investigated the topographical organization of pulvino-cortical functional connectivity. Ideally, one would like to describe pulvino-cortical functional connectivity at single-voxel resolution. However, this poses important visualization issues. In order to circumvent this, we decided for a more approximative approach. Specifically, we manually segmented the main pulvinar nuclei (Fig. 1, left panels), and we subdivided them in 2 mm-thick slices along the latero-medial (Fig, 1, top panel), ventro-dorsal (Fig. 1, middle panel), and antero-posterior axes (Fig. 1, bottom panel). We computed the functional connectivity of each of these slices with the rest of the brain and projected the winner-take-all voxels of this functional connectivity analysis on the corresponding semi-inflated brains. We used the same color code as inFigure 1(seeSection 2; please note that only ipsilateral pulvino-cortical connectivity is considered in the figures except SupplementaryFig. S7). We first report a global organization of pulvino-cortical functional connectivity, which is observed at whole-brain level and that can be precisely captured along the different axes described inFigure 1. We then report local functional connectivity patterns that are observed in most individuals, though with some degree of inter-individual variability.

### 3.1 Global pulvino-cortical functional connectivity gradients

**Fig. 2.**
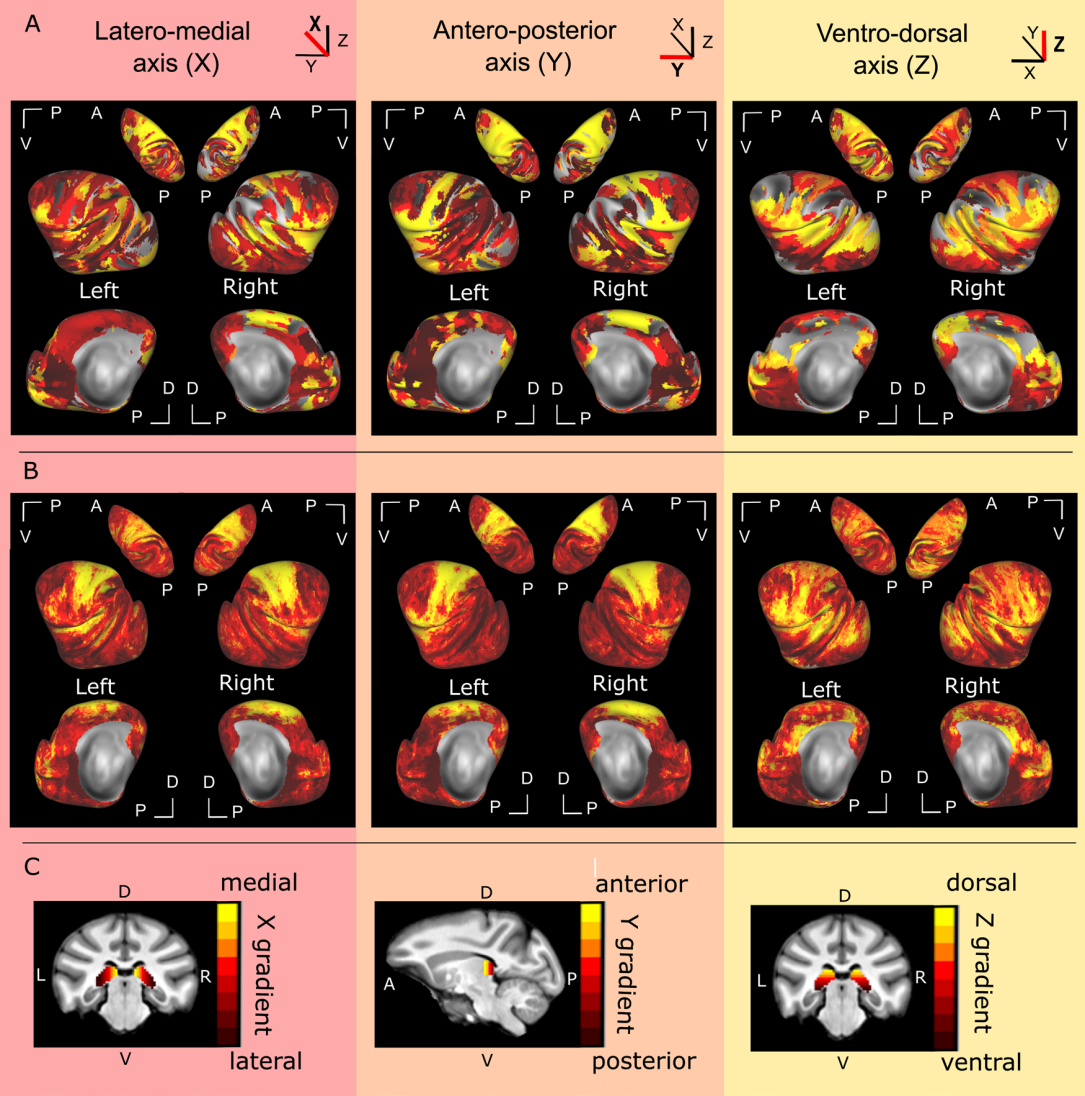
Global functional connectivity gradient of the pulvinar with the cortex**.**The pulvinar is subdivided into slices of 2 mm thickness. These slices are used as seeds for a seed-to-whole-brain functional connectivity analysis. A winner-take-all procedure is then applied to associate (color code) each cortical voxel with the pulvinar slice it maximally correlates with. Only ipsilateral correlations are presented. (A) Winner-take-all functional connectivity map for a single subject (LA) for the medial and lateral representations and (FI) for the representation from the top**.**(B) Mean winner-take-all functional connectivity maps across the 10 monkeys. (C) Pulvinar slice seeds used in the functional connectivity analysis, for each of the analysis axes of interest. Details are provided inFigure 1.

The distinct functional connectivity patterns of the pulvinar with the cortex along the three aforementioned axes are presented inFigure 2, for an individual subject (Fig. 2A) and averaged over all subjects (seeSection 2,Fig. 2B). Individual subjects are present in SupplementaryFigure S3. The correlation maps represent the winner-take-all correlations between each voxel of the cortex and a unique pulvinar slice. This analysis is somewhat misleading as any given cortical voxel can be functionally connected to multiple pulvinar slices, but with varying functional connectivity strength. Lowering the criterion of maximal functional connectivity (winner-take-all approach) changed only marginally the reported observations (SupplementaryFigs. S4 and S5). This analysis leads to the description of complex functional connectivity gradients between the pulvinar and the cortex. It is important to note that due to the shape of the pulvinar, these gradients are not independent one from the other.

#### 3.1.1 Latero-medial gradient

Both for a single subject (Fig. 2A, left panel, see also SupplementaryFig. S3) and the group data (Fig. 2B, left panel), the sensorimotor cortex, the premotor cortex, the dorsal cingulate cortex, and the insula are robustly correlated with the medial pulvinar. This is in line with the anatomical literature (Kaas & Lyon, 2007;Mufson & Mesulam, 1984;Padberg et al., 2009;Rosenberg et al., 2009;Wilke et al., 2018;Yeterian & Pandya, 1988). The medial pulvinar also shows reproducible functional connectivity with the inferior prefrontal cortex and the medio-posterior part of the superior temporal sulcus as well as a patchy connectivity with the extrastriate cortex. In contrast, the lateral pulvinar has the strongest functional connectivity with the anterior part of the superior temporal sulcus as well as with the dorsal lateral prefrontal cortex. These connectional features are better visible for individual brains and become less prominent in the group data, likely due to inter-individual differences. Note that this latero-medial parcellation of the pulvinar is partially overlapping with the anterior-posterior and ventral-dorsal parcellations, due to the specific 3D shape of the pulvinar.

#### 3.1.2 Antero-posterior gradient

Similarly to the previous gradient, both on the single exemplar brain (Fig. 2A, middle panel, see also SupplementaryFig. S3) and the group data (Fig. 2B, middle panel), the sensorimotor cortex, the premotor cortex, the dorsal cingulate cortex, and the insula are functionally correlated with the anterior pulvinar (yellow) which is in line with the anatomical literature (Kaas & Lyon, 2007;Mufson & Mesulam, 1984;Padberg et al., 2009;Rosenberg et al., 2009;Wilke et al., 2018;Yeterian & Pandya, 1988). On the other hand, occipital areas and a large part of the posterior medial wall of the cortex are functionally more connected with the posterior pulvinar. Interestingly, the most anterior part of the brain, that is, the prefrontal and orbitofrontal cortex, shows stronger connectivity with the posterior and central parts of the pulvinar than with its anterior part, thus disrupting the antero-posterior gradient. Specifically, the prefrontal cortex correlates more with slices containing more medial pulvinar than the anterior pulvinar, whether analyzing functional connectivity along the ventro-dorsal or the antero-posterior gradient (see SupplementaryFig. S1).

#### 3.1.3 Ventro-dorsal gradient

In both single-subject (Fig. 2A, right panel, see also SupplementaryFig. S3) and group data (Fig. 2B, right panel), strong functional connectivity can be observed between the dorsal part of the pulvinar and dorsal extrastriate cortex, the ventral part of the intraparietal sulcus, dorsal lateral sulcus, ventral sensorimotor cortex, and ventral prefrontal cortex (yellow). More ventral pulvinar regions, on the other hand, are functionally more connected with the ventral occipital and temporal cortex. This ventro-dorsal functional gradient of the pulvino-cortical functional connectivity is also in agreement with the literature (Arcaro et al., 2015,^2018^;Dominguez-Vargas et al., 2017;Shipp, 2003;Wilke et al., 2010).

Overall, looking at the global cortical patterns, pulvino-cortical connectivity predominantly follows the ventro-dorsal and the antero-posterior gradient (except for the most anterior part of the cortex). In order to investigate the degree of homogeneity of the different gradients of connectivity, we performed a spatial correlation analysis between the “winner take all” spatial maps from the different subjects. This analysis shows that the correlation maps were significantly correlated across subjects (p < 0.001), indicating a consistency for the pulvino-cortical connectivity across subjects despite inter-individual differences (Spearman correlation coefficient (ρ):*antero-posterior gradient*: mean of both hemispheres = 0.46;*latero-medial gradient*: mean = 0.38;*ventro-dorsal gradient*: mean = 0.4). This correlation was highest along the antero-posterior gradient, suggesting that the antero-posterior connectivity gradient from the premotor cortex to the occipital cortex, and its disruption in the prefrontal and orbitofrontal cortex was similar across individuals (Friedman non-parametric test, X2(38) = 22.9, p < 0.001; post-hoc: antero-posterior axis vs. latero-medial axis: p = 0.0001; antero-posterior axis vs. ventro-dorsal axis: p = 0.0019; latero-medial axis vs. ventro-dorsal axis: p = 0.5938).

### 3.2 Local connectivity gradients and multiple functional pulvino-cortical projection fields

While common global functional connectivity patterns can be identified between the pulvinar and the cortex characterized by both a ventro-dorsal and antero-posterior gradient, inter-individual differences do exist and some regions such as the prefrontal cortex escape these global functional connectivity patterns. This can be considered either noise, or a signature of specific local functional connectivity patterns between the pulvinar and the cortex. We addressed the latter hypothesis. InFigure 3, we focus on the functional connectivity of the pulvinar within restricted regions of the brain to investigate local connectivity gradients and to identify possible common functional connectivity patterns across different regions. Specifically, we focused on seven sulci and selected the functional connectivity axis of the pulvinar that resulted in statistically most robust topographic patterns (decision criterion: significant correlation in both hemispheres reflecting pulvino-cortical connectivity gradient along the same anatomical orientation, SupplementaryTable S1).

**Fig. 3.**
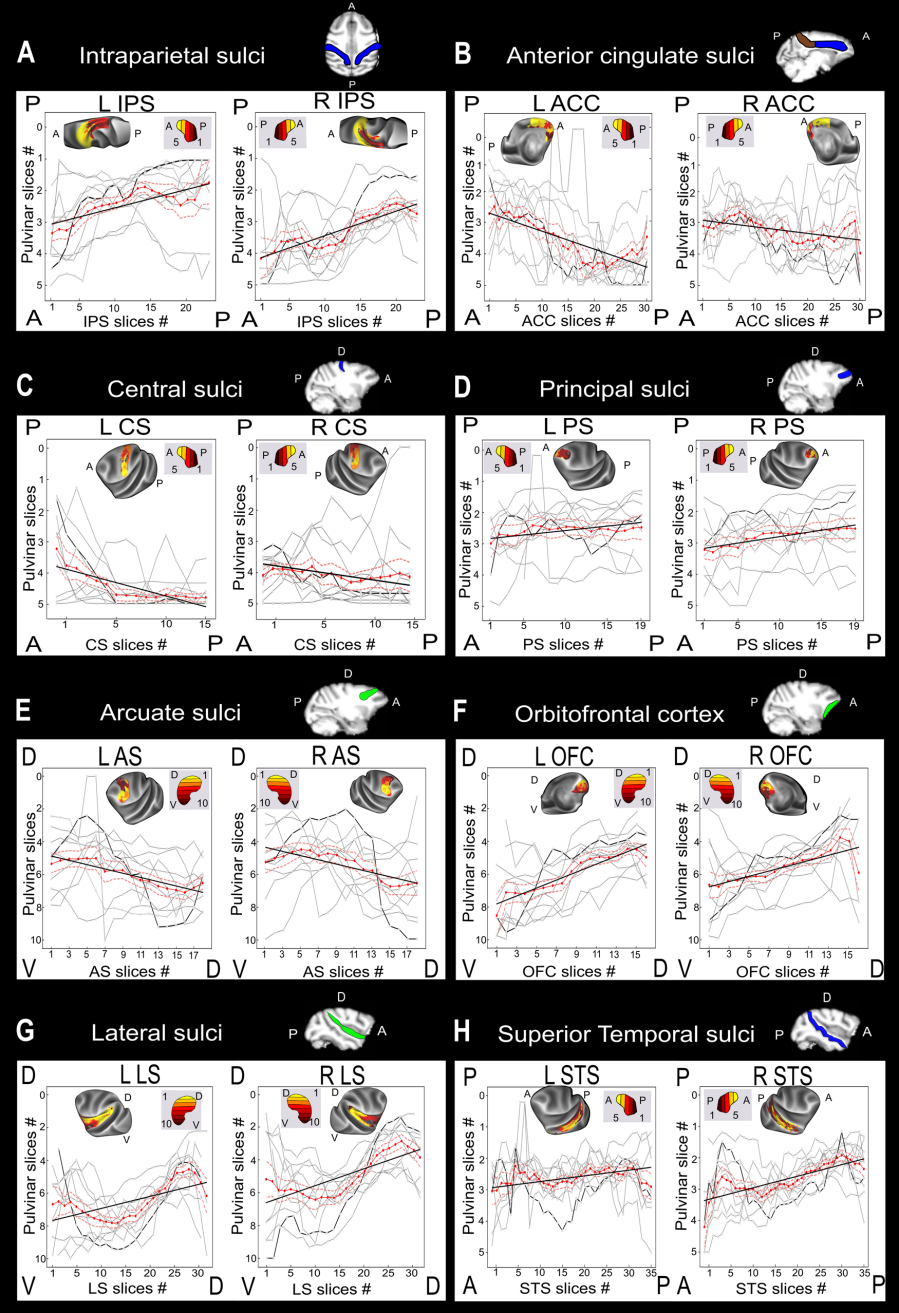
Local functional connectivity gradients of the pulvinar with specific brain regions. The pulvinar connectivity is retrieved for selected sulci: the intraparietal sulcus (A), anterior cingulate cortex (B), central sulcus (C), principal sulcus (D), arcuate sulcus (E), orbitofrontal cortex (F), lateral sulcus (G), and the superior temporal sulcus (H). Only ipsilateral correlations are presented. Sulci correlating with the pulvinar along the ventro-dorsal axis are colored in green and in blue when the correlation follows the antero-posterior axis. These correlations are retrieved for each subject (grey) and the mean of all the subjects (red) together with linear regressions (black). Note that only one anatomical axis is significant for both hemispheres for each sulcus. The illustrating inset maps are masked from individual maps (LA); however, the IPS (panel A) was created from another individual map (FI) because monkey LA presents a lack of connectivity in this sulcus. The connectivity gradients for these individuals are represented in darker gray.

#### 3.2.1 Intraparietal sulcus

The intraparietal sulcus, which is mainly oriented along an antero-posterior axis, shows a functional connectivity pattern with the pulvinar, which follows an antero-posterior gradient, that is, a gradient organized along the same axis as the sulcus (L IPS: p = 19.9 x 10^-7^; r = 0.33; R IPS: p = 5.5 x 10^-14^; r = 0.463,Fig. 3A, SupplementaryTable S1).

#### 3.2.2 Cingulate sulcus

The anterior cingulate cortex, which is also oriented along an antero-posterior axis, shows a pattern of functional connectivity with the pulvinar, again following an antero-posterior gradient, that is, a gradient organized along the same axis as the sulcus (L ACC: p = 2.4 x 10^-19^; r = 0.46; R ACC: p = 0.0002; r = 0.285,Figure 3B, SupplementaryTable S1). However, contrary to the IPS, the ACC shows inverted connectivity with the pulvinar, whereby its most anterior part correlated more with the posterior sectors of the pulvinar. This indicates that the spatial organization of functional pulvinar projections to the cortex is not an artefact of distance from the nucleus. In comparison, the posterior cingulate cortex (PCC) does not present a clear orientation for its connectivity with the pulvinar (see SupplementaryTable S1).

#### 3.2.3 Central sulcus

The central sulcus, which is also oriented along an antero-posterior axis, shows a functional connectivity pattern with the pulvinar, again following an antero-posterior gradient, that is, a gradient organized along the same axis as the sulcus (L CS: p = 1.36 x 10^-12^; r = 0.52; R CS: p = 0.0004; r = 0.27,Fig. 3C, SupplementaryTable S1).

#### 3.2.4 Prefrontal cortex

In the prefrontal cortex, the tip of the anterior principal sulcus was consistently connected with anterior pulvinar slices, and the pattern of functional connectivity gradually shifted towards more posterior pulvinar slices more posteriorly along this sulcus (L PS: p = 0.04; r = 0.36; R PS: p = 0.001; r = 0.23,Fig. 3D, SupplementaryTable S1). The arcuate sulcus showed a reversed ventro-dorsal functional connectivity pattern with the pulvinar, with the pulvinar dorsal part correlating with the ventral most part of the arcuate sulcus (L AS: p = 2.336 x 10^-6^; r = 0.46; R AS: p = 1.13 x 10^-3^; r = 0.35,Fig. 3E, SupplementaryTable S1). The orbitofrontal cortex demonstrated a ventro-dorsal connectivity pattern with the pulvinar (L OFC: p = 7.06 x 10^-3^; r = 0.51; R OFC: p = 9.2 x 10^-13^; r = 0.51,Fig. 3F, SupplementaryTable S1). These diverse functional connectivity patterns in the prefrontal cortex might explain why this region does not demonstrate a clear global functional connectivity pattern with the pulvinar.

#### 3.2.5 Lateral sulcus

In the LS, the dorsal tip of the sulcus is mostly connected with dorsal pulvinar, and functional connectivity consistently shifted towards more ventral pulvinar slices as we moved anteriorly along the sulcus. This linear trend was disrupted when reaching the dorsal insula, anteriorly. This region expresses strong functional connectivity with the dorsal and medial pulvinar slices. A linear regression was performed to investigate the orientation of the pulvinar functional connectivity along these sulci. For both the left and right LS, the functional connectivity significantly followed a ventro-dorsal gradient (L LS: p = 6.8 x 10^-12^; r = 0.4; R LS: p = 8.87 x 10^-14^; r = 0.44,Fig. 3G, SupplementaryTable S1).

#### 3.2.6 Superior temporal sulcus

In the STS, the dorsal part of the sulcus is functionally connected most strongly with the dorsal pulvinar, and functional connectivity patterns consistently shifted to more ventral pulvinar slices as we moved anteriorly along the sulcus. This linear trend was disrupted when reaching the ventral part STS that shows a preferential connectivity with the dorsal pulvinar. This functional connectivity is in line with the literature showing functional connectivity between the most anterior AM face patch and the dorsal pulvinar (Schwiedrzik et al., 2015). Additionally, the medial pulvinar is functionally connected with the caudal part of the STS and the lateral pulvinar with the rostral part of the STS. Thus, a dual functional connectivity gradient can be identified in this sulcus. As the STS shows this dual gradient along the ventro-dorsal axis (see SupplementaryFig. S6), the linear regression along this axis was not significant (L STS: p = 0.23; r = 0.06; R STS: p = 0.11; r = 0.01), though the linear regression reached significance along the antero-posterior axis (L STS: p = 0.007; r = 0.02; R STS: p = 1.06 x 10^-15^; r = 0.4,Fig. 3H, SupplementaryTable S1).

Overall, this suggests that, beyond the global gradients described in the previous sections, multiple pulvinar projection fields can be identified at cortical level. These projection fields, though systematic, are subject to substantial inter-individual variability and thus disappear on group average analyses. This observation, however, is expected to have profound implications on our understanding of the functional interactions between the pulvinar and the cortex.

## 4 Discussion

Our results provide fine-grained functional connectivity patterns of the pulvinar with the cortex at several spatial scales. First, we show a global topographical connectivity pattern that can be captured along ventro-dorsal and antero-posterior pulvinar gradients and to a lesser extent along a medio-lateral gradient. We additionally describe more refined local functional connectivity patterns that capture multiple cortical pulvinar projection fields.

**Fig. 4.**
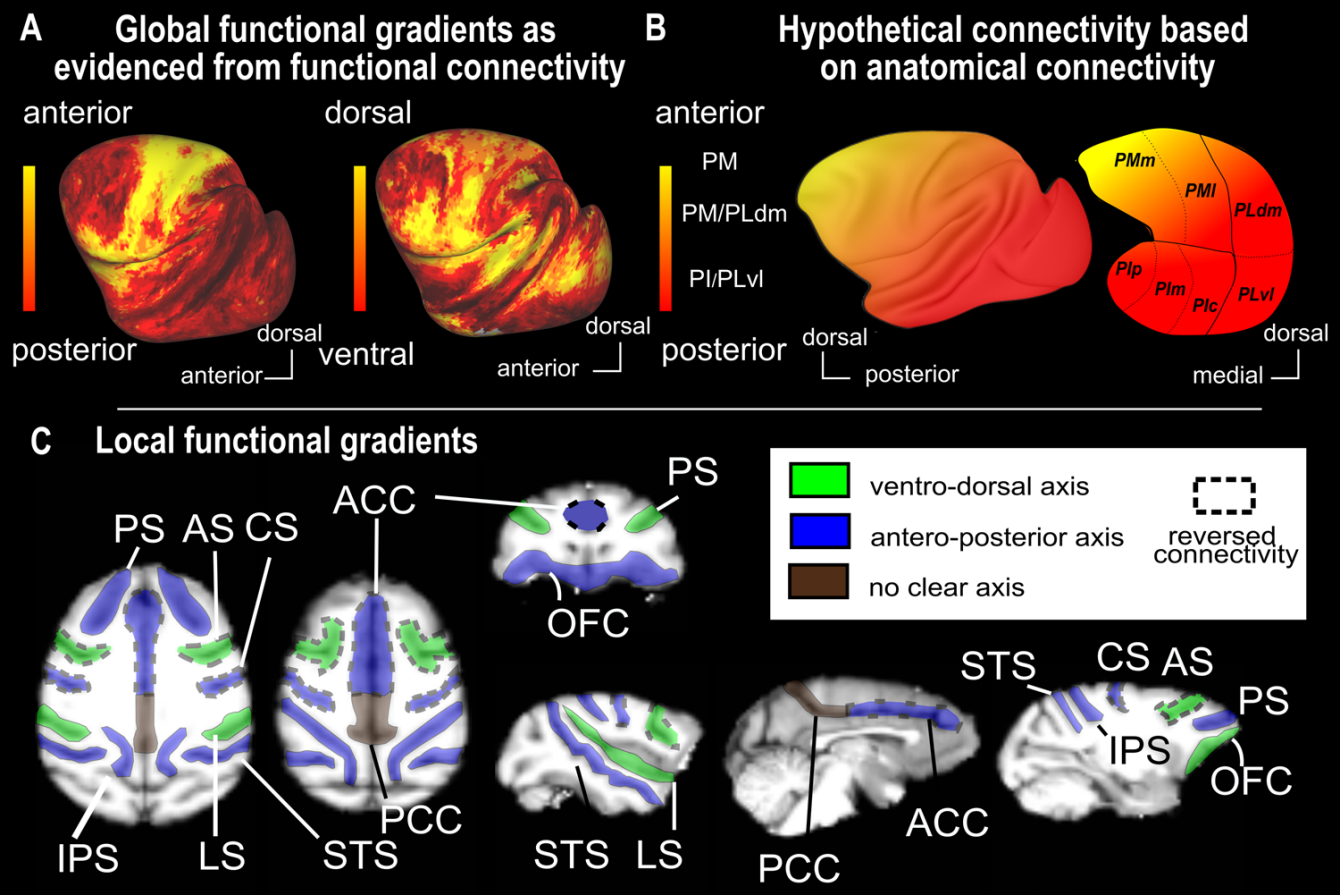
Summary of the global and local pulvino-cortical connectivity orientation. (A) Results of the seed-to-whole-brain functional connectivity analysis for the antero-posterior gradient (left) and the ventro-dorsal gradient (right), fromFigure 2. (B) Predicted mixed orientation of the functional connectivity based on anatomical literature (based on the reviewFroesel et al., 2021). (C) Summary of the local pulvino-cortical connectivity orientation. Dashed lines represented reversed connectivity pattern with the anatomical axis (ACC, AS and CS). PS: principal sulcus; ACC: Anterior cingulate sulcus; PCC: Posterior cingulate sulcus; OFC: orbitofrontal cortex; CS: central sulcus; AS: Arcuate sulcus; LS: lateral sulcus; STS: Superior temporal sulcus; IPS: intraparietal sulcus.

The goal of this work was to characterize more precisely the functional connectivity of the pulvinar with the cortex, in order to anatomically constrain its role in a variety of cognitive functions. In monkey fMRI studies, the cohort size is often a strong limitation (Milham et al., 2020,^2022^). Here, we characterized pulvino-cortical functional connectivity patterns at rest, relying on 10 awake fixating macaques. We confirm previous observations that the pulvinar has widespread connections with the cortex supporting its involvement in multiple cognitive functions (Barron et al., 2015;Froesel et al., 2021). Additionally, we show that the functional connectivity patterns we revealed resemble, at least partially, previously described anatomical connectivity, thus describing a global connectivity gradient along both the antero-posterior and ventro-dorsal axes (Fig. 4Apresents the results of functional connectivity and 4B presents the functional connectivity as predicted by anatomical studies). Indeed, anatomical studies predict a high functional connectivity between the inferior and ventro-lateral pulvinar subregions and early visual cortical areas and posterior parietal and temporal cortical regions. Likewise, the medial and dorso-lateral pulvinar subdivisions are expected to connect mostly with intraparietal, prefrontal, STS, and insula regions. These findings are in line with the neuronal recording and microstimulation literature (Kagan et al., 2021). In contrast, anatomical studies predict that anterior pulvinar connects with the anterior part of the brain. This does not fit with the functional connectivity reported here. This is most probably due to the fact that anatomical studies often considered the medial, lateral, and inferior pulvinar subdivisions excluding the most anterior part of the pulvinar and making the comparison with our current study not directly straightforward. We confirm the functional connectivity of the medio-dorsal pulvinar with the medial bank of the IPS. The highly robust connectivity between the anterior part of the pulvinar and the sensorimotor and premotor cortex is also in agreement with previous studies (Kaas & Lyon, 2007;Mufson & Mesulam, 1984;Padberg et al., 2009;Rosenberg et al., 2009;Wilke et al., 2018;Yeterian & Pandya, 1988). The disruption of the anterior-posterior connectivity gradient in the prefrontal cortex is therefore also predicted by the literature, the anterior pulvinar being more connected with the motor and premotor regions whereas the medial pulvinar is described as interacting with the more anterior region of the brain such as the FEF, the DLPFC, or the OFC (Froesel et al., 2021). Overall, the reported functional connectivity in the present work thus reliably reproduces observations collected with a diversity of methods and sets the stage for the analysis of pulvino-cortical functional connectivity at a more resolved spatial granularity.

A major result of this work is that beyond the global connectivity gradients described above, multiple local pulvinar projections fields can be identified in multiple cortical regions. We focused on the sulci level and identified gradients of connectivity between the pulvinar and the sulci but also some within-sulci gradients, suggesting a notion of multiple projection fields of the pulvinar onto the cortex. The organization of these pulvinar projections fields is reproduced across animals and hemispheres. Such local topographical organization of pulvinar connectivity has been occasionally reported in specific cortical regions such as the superior temporal sulcus or MT (Grimaldi et al., 2016;Mundinano et al., 2019), but to the best of our knowledge, never in such detail as here. We propose that this is a fundamental principle of pulvino-cortical connectivity and that a better description of these connectional principles will be crucial to fully understand the function of pulvino-cortical connections, as well as the role of the pulvinar in cognition. This organization, although preserved across individuals, is susceptible to some degree of inter-individual variability.

The local pulvinar projection fields are summarized inFigure 4C. Using a color code, we indicated the dominant orientation of the functional connectivity patterns between the pulvinar and the different sulci. The orientation of the functional connectivity gradients is dominated by ventro-dorsal and antero-posterior pulvinar projections, often matching the dominant orientation of the sulci. It is interesting to highlight that the sulci from the posterior part of the brain tend to follow the antero-posterior axis, matching the global gradient, while the sulci from the prefrontal region show more diverse preferred orientations of the connectivity. Please also note that while the color reflects dominant functional connectivity orientation, it does not indicate the polarity of the projections, such that the anterior pulvinar can correlate with the anterior part of the sulcus and the posterior pulvinar can correlate with the posterior part of the sulcus, or the other way round, such that the anterior pulvinar can correlate with the posterior part of the sulcus and the posterior pulvinar can correlate with the anterior part of the sulcus. For example, the anterior cingulate cortex has a reversed antero-posterior pulvinar mapping relative to the other sulci dominated by antero-posterior pulvinar projections. Indeed, the most anterior part of the cingulate sulcus correlates with the most posterior part of the pulvinar and the most posterior part of the cingulate sulcus correlates with the most anterior part of the pulvinar. This reversed connectivity pattern is also found in the central sulcus and the arcuate sulcus, possibly suggesting a very specific and more refined organization of the connectivity between the pulvinar and the anterior part of the cortex as compared to the posterior part of the brain. The dual functional connectivity gradient observed within the STS is yet another expression of the complex organization of the pulvino-cortical functional connectivity. While this sulcus follows a global antero-posterior gradient of functional connectivity with the pulvinar two ventro-dorsal gradients are observed, thus determining the pulvinar projections field along the STS (SupplementaryFig. S6). We hypothesize that this rich connectivity allows a very refined interaction between the subcortical nucleus and the superior temporal sulcus. Fine multimodal studies combining high-resolution fiber tracking (Tounekti et al., 2018) and voxel-based functional connectivity analysis are expected to clarify the origin of these multiple cortical pulvinar projection fields.

The description of these functional gradients characterizing the local cortical pulvinar projection fields does not exclude the possibility that any given cortical region can receive input from multiple pulvinar sectors. For example, a correlation of the STS and others cortical regions can be described with both ventral and dorsal pulvinar (data not shown), confirming the microstimulation results recently reported byKagan et al. (2021). In addition, the functional connectivity between any of these regions and the pulvinar can reflect, like is the case with microstimulation-evoked effective connectivity (as microstimulation elicits cortical activations in the contralateral hemisphere, Kagan et al., 2021), both direct (monosynaptic) and indirect (polysynaptic) pulvinar connectivity to the cortex. Speaking to this, the contralateral pulvinar connectivity maps are remarkably similar to the ipsilateral maps (SupplementaryFig. S7) as well as the absence of evidence for monosynaptic pulvinar connections to the contralateral hemisphere. These projections from multiple pulvinar nuclei in specific brain regions have been a core argument in support of the role of the pulvinar as a modulator (Benarroch, 2015;Saalmann & Kastner, 2011). The pulvinar can thus be a part of several networks and can be recruited differentially as a function of sensory modality or the ongoing cognitive demand (Froesel et al., 2024), thus supporting cognitive flexibility. As a result, another important aspect to keep in mind is that the winner-take-all maps we report here from monkeys at rest could be different during active tasks involving either sensory stimulation or the production of complex behaviors. We hypothesize that at rest, the winner-take-all maps will follow more closely the anatomical structural connectivity (Honey et al., 2007,^2009^), while in contrast, during systemic localizer studies (Russ et al., 2021) or active behavior, the correlations would be more pronounced in specific functional networks, the functional connectivity with other subregions being possibly attenuated. Last, when interpreting the current observations, technical aspects need to be kept in mind in addition to the interpretation of the winner-take-all approach and the possibility of overlapping connectivity fields from different pulvinar subregions. Some of these aspects pertain to the spatial resolution of the data and associated possible partial volume effects, possibly obscuring a finer-grained anatomical organization. Other aspects relate to the fact that local gradients average possibly diverse functional connectivity patterns orthogonally to the sulci, as the two banks and fundus of the different sulci may express distinct connectivity patterns with the pulvinar. Investigating these points would be a logical follow-up of the present study. Other considerations yet include the very measure of functional connectivity from resting-state data used here, that lacks directionality information and that might occasionally describe second-order rather than first-order connectivity. Fine-grained multimodal studies combining rs-fMRI, DTI, and anatomical tracing will be needed to resolve these issues.

## 5 Conclusion

Two scales of functional connectivity between the pulvinar and the cortex are described. A global topographical functional connectivity pattern can mainly be captured along the antero-posterior and ventro-dorsal axes, and multiple local topographically organized pulvinar projections fields in multiple cortical regions such as the lateral sulcus, the superior temporal sulcus, the anterior cingulate cortex, and the intraparietal sulcus. In spite of the inter-individual differences, the local functional gradients are consistently described across individuals and in both hemispheres and follow the anatomical orientation of the sulci. We propose that these multiple pulvinar projection fields correspond to a fundamental principle of pulvino-cortical connectivity and that a better understanding of this organization will help clarifying the function of pulvino-cortical connectivity and functions.

## Supplementary Material

Supplementary Material

## Data Availability

Data and code are available upon request.
