# Supplementary material for: Multiple focal pulvinar projection fields in the macaque cortex
Source: Imaging Neurosci (Camb). 2024 Jun 26;2:imag-2-00202. doi: 10.1162/imag_a_00202 (PMC12272232; doi:10.1162/imag_a_00202)
Supplement: Supplementary Material [file imag_a_00202-supp.pdf]

Supplementary data

ACC	L ACC X	r value	0,125	R ACC X	r value	0,15
		p value	0,34		p value	0,2
		Lin. Coef.	0,0324		Lin. Coef.	0,077
	L ACC Y	r value	0,46	R ACC Y	r value	0,285
		p value	2,4E-19		p value	0,0002
		Lin. Coef.	-0,06		Lin. Coef.	-0,018
	L ACC Z	r value	0,105	R ACC Z	r value	0,5774
		p value	0,2		p value	***0,0077***
		Linear Coefficient	0,056		Lin. Coef.	0,0414
AS	L AS X	r value	0,54	R AS X	r value	0,33
		p value	***2,09E-15***		p value	***1,37E-06***
		Lin. Coef.	0,1188		Lin. Coef.	-0,0639
	L AS Y	r value	0,25	R AS Y	r value	0,07
		p value	**0,0020**		p value	0,32
		Lin. Coef.	-0,05		Lin. Coef.	-0,01
	L AS Z	r value	0,46	R AS Z	r value	0,35
		p value	2,36E-08		p value	1,13E-06
		Lin. Coef.	-0,13		Lin. Coef.	-0,1094
CS	L CS X	r value	0,51	R CS X	r value	0,28
		p value	***2,52E-19***		p value	***1.8E-06***
		Lin. Coef.	-0,16		Lin. Coef.	0,0618
	L CS Y	r value	0,52	R CS Y	r value	0,27
		p value	1,36E-12		p value	0,004
		Lin. Coef.	-0,1		Lin. Coef.	-0,0136
	L CS Z	r value	0,18	R CS Z	r value	0,14
		p value	***0.007***		p value	***0,03***
		Lin. Coef.	-0,008		Lin. Coef.	0,0388
STS	L STS X	r value	0,022	R STS X	r value	0,175
		p value	0,68		p value	***0,01***
		Lin. Coef.	-0,005		Lin. Coef.	0,023
	L STS Y	r value	0,13	R STS Y	r value	0,4
		p value	0,007		p value	1,06E-15
		Lin. Coef.	0,02		Lin. Coef.	0,028
	L STS Z	r value	0,06	R STS Z	r value	0,08
		p value	0,23		p value	0,11
		Lin. Coef.	0,001		Lin. Coef.	0,01
IPS	L IPS X	r value	0,0038	R IPS X	r value	0,04
		p value	0,93		p value	0,51

		Linear Coef	-0,02		Linear Coef	-0,0106
	L IPS Y	r value	0,3351	R IPS Y	r value	0,46
		p value	1,9E-07		p value	5,53E-014
		Lin. Coef.	0,029		Lin. Coef.	0,05
	L IPS Z	r value	0,059	R IPS Z	r value	0,14
		p value	0,47		p value	0,09
		Lin. Coef.	0,007		Lin. Coef.	0,045
LS	L LS X	r value	0,055	R LS X	r value	0,38
		p value	0,4739		p value	***1,03E-0705***
		Lin. Coef.	-0,02		Lin. Coef.	0,1075
	L LS Y	r value	0,0686	R LS Y	r value	0,104
		p value	0,21		p value	0,054
		Lin. Coef.	0,007		Lin. Coef.	0,008
	L LS Z	r value	0,4	R LS Z	r value	0,44
		p value	6,8E-122		p value	8,87E-14
		Lin. Coef.	0,1		Lin. Coef.	0,109
OFC	L OFC X	r value	0,15	R OFC X	r value	0,067
		p value	***0.01-06***		p value	0,32
		Lin. Coef.	0,038		Lin. Coef.	-0,00633
	L OFC Y	r value	0,02	R OFC Y	r value	0,164
		p value	0,69		p value	***0,017***
		Lin. Coef.	-0,01		Lin. Coef.	0,01986
	L OFC Z	r value	0,514	R OFC Z	r value	0,51
		p value	7,06E-		p value	9,28E-13
		Lin. Coef.	0,17		Linear	0,17
PS	L PS X	r value	0,122	R PS X	r value	0,06
		p value	0,1019		p value	0,38
		Lin. Coef.	0,05		Linear	0,01
	L PS Y	r value	0,36772	R PS Y	r value	0,23
		p value	0,042		p value	0.001
		Lin. Coef.	0,0173		Lin. Coef.	0,042
	L PS Z	r value	0,108	R PS Z	r value	0,07
		p value	0,283		p value	0,406
		Lin. Coef.	-0,014		Lin. Coef.	-0,043
PCC	L PCC X	r value	0,45	R PCC X	r value	0,105
		p value	***0,0002***		p value	0,352
		Lin. Coef.	0,2662		Lin. Coef.	-0,0694
	L PCC Y	r value	-0,0267	R PCC Y	r value	0,088
		p value	0,714		p value	0,18
		Lin. Coef.	0,015		Lin. Coef.	-0,016
		r value	0,128		r value	0,013

	L PCC Z	p value	0,09	R PCC Z	p value	0,866
		Lin. Coef.	0,052		Lin. Coef.	0,0037

Supplemental Table 1: Detailed statistics of the linear regression analysis. Significative correlations are indicated in bold italic. Anatomical orientation presenting a significant correlation in both hemispheres with the same orientation for any given sulcus is indicated in bold red and highlighted against a grey background. X: latero-medial axis. Y: antero-posterior axis. Z: ventro-dorsal axis.

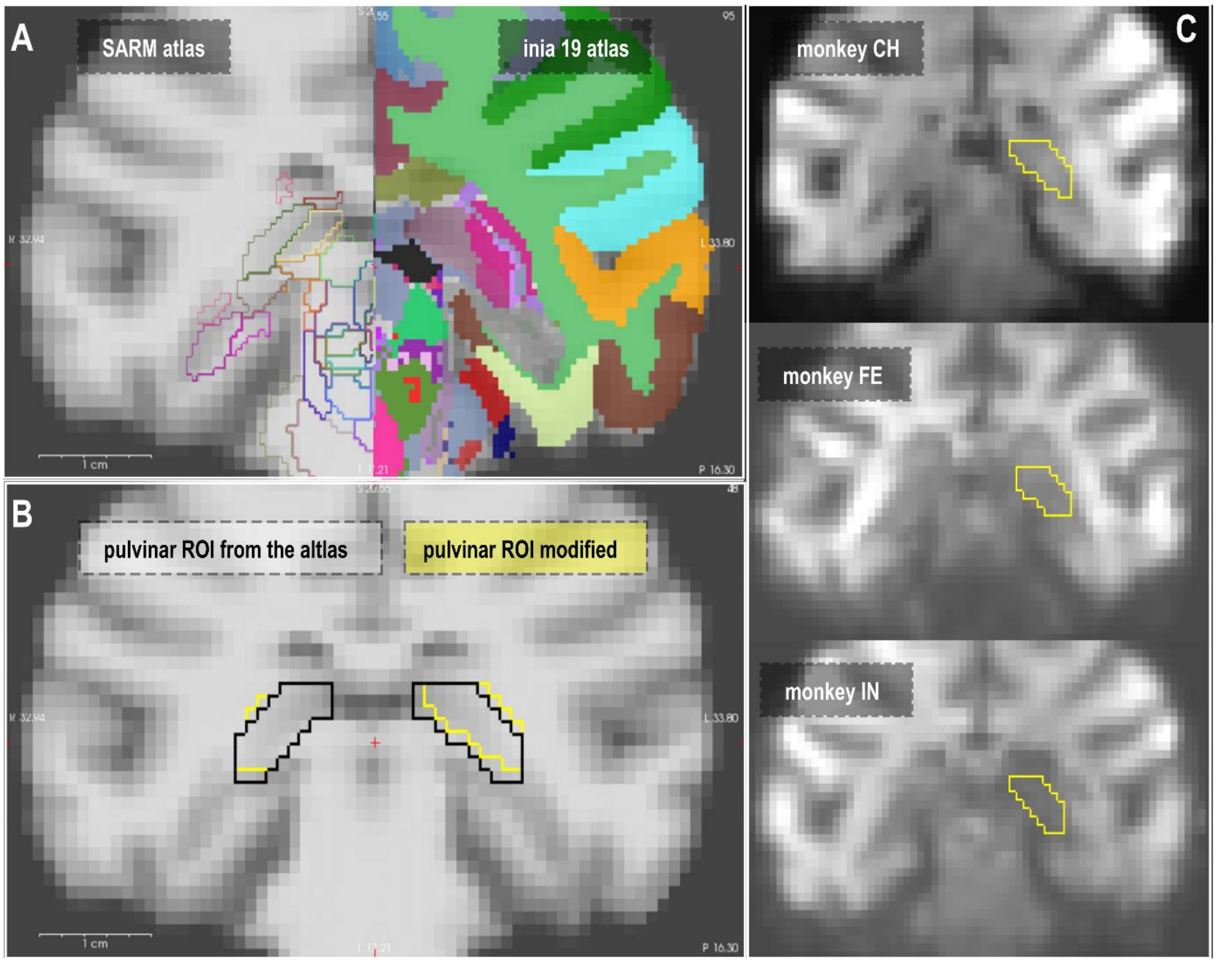

Figure S1: Definition of subject specific pulvinar ROIs. A) SARM and Inia 19 NeuroMaps Atlas were coregistered on the anatomical common space. B) Atlas-defined pulvinar ROIs (black outlined) and modified ROIs by hand (yellow outline). C) Morphologic disparities of the monkeys. Three functional examples are presented. Left: anatomical coronal section through the pulvinar. Right: anatomical coronal section through the pulvinar with hand drawn pulvinar contours in yellow. All sections are taken at -11mm from the anterior commissure after coregistration on the anatomical common space.

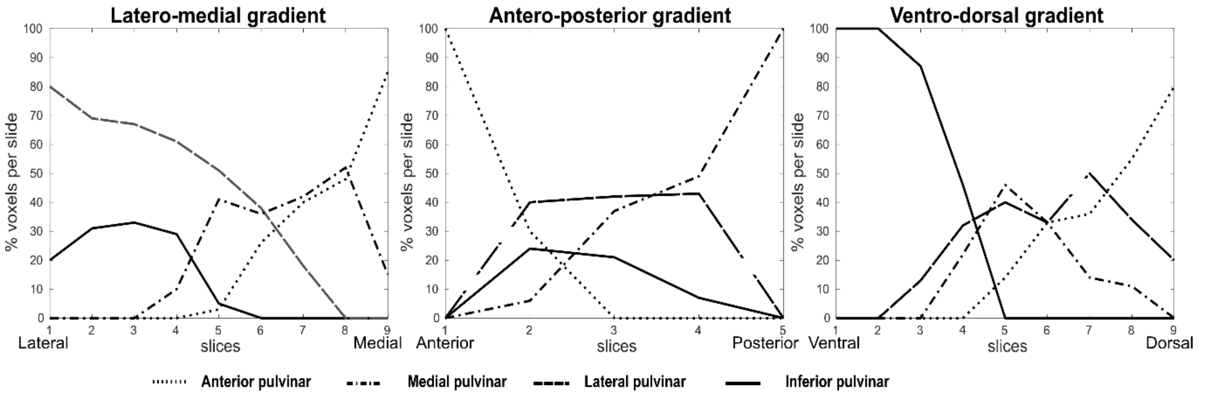

Figure S2: Proportion of voxels belonging to each pulvinar sub parts, i.e. anterior, medial, lateral and inferior pulvinar, per slice.

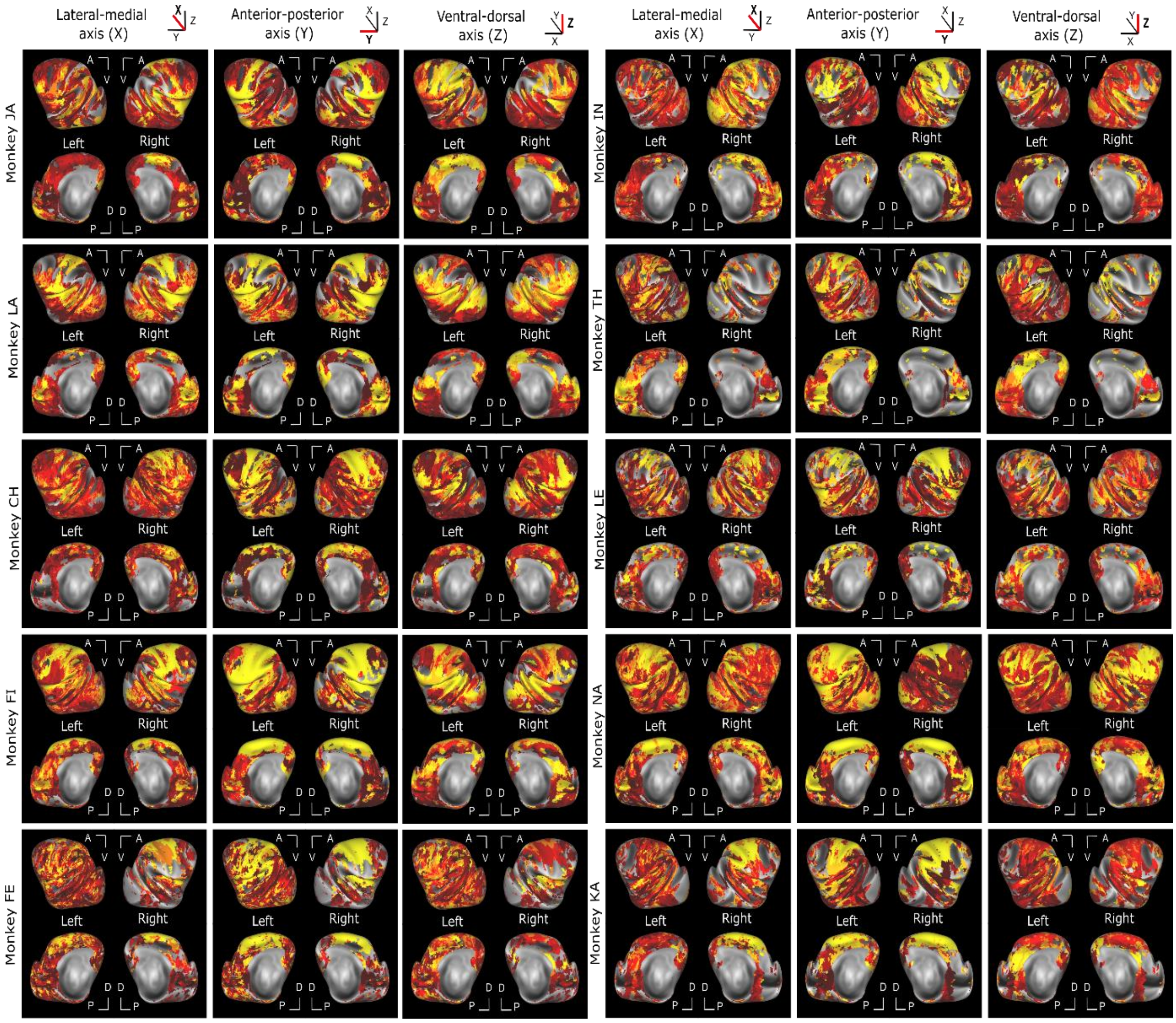

Figure S3: Winner take all functional connectivity maps for each individual monkey. All else as in figure 2. Only ipsilateral correlations are presented.

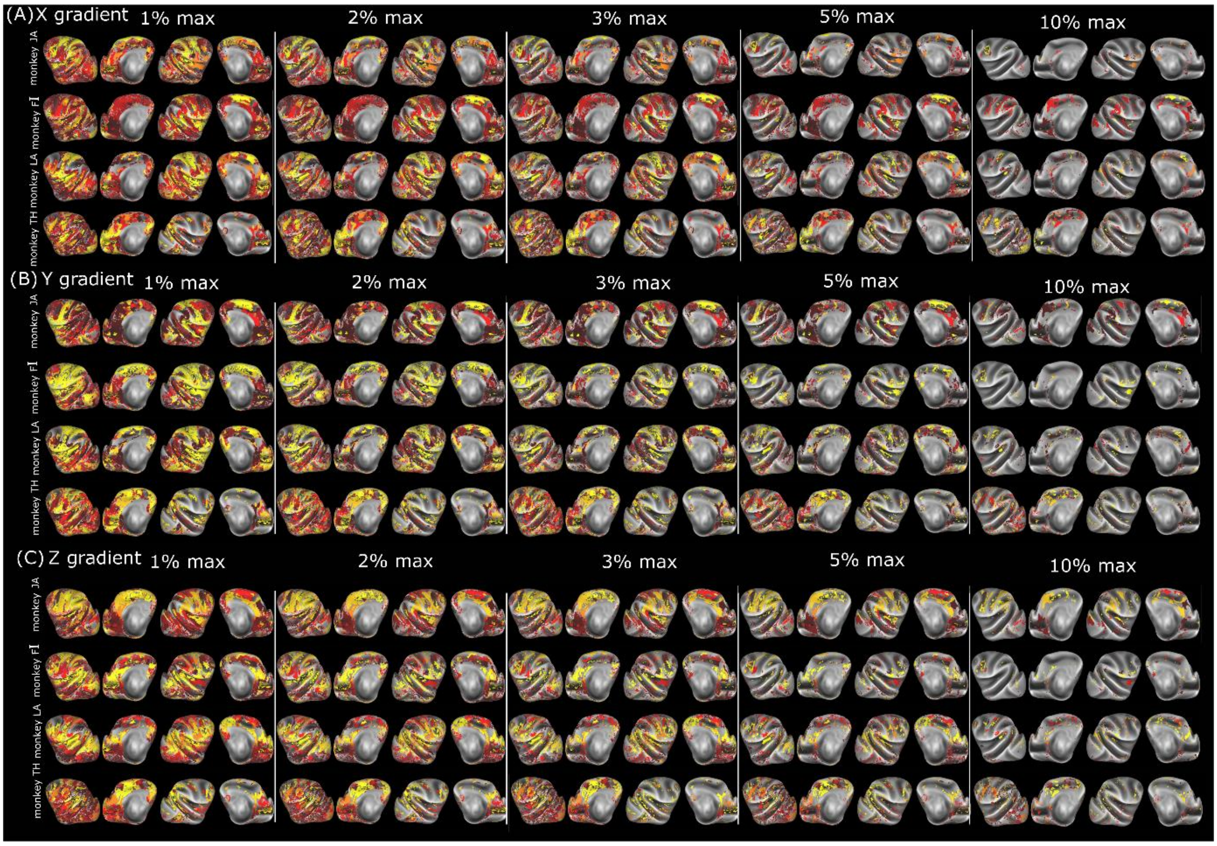

Figure S4: Winner take all functional connectivity maps for individual monkeys as a function of percent of correlation strength distance to the second best correlating voxel. The maps are the winner take all functional connectivity maps displaying only the correlation that are 1, 2 ,3, 5 or 10% higher than the second best correlation. Four exemplar monkeys. Only ipsilateral correlations are presented.

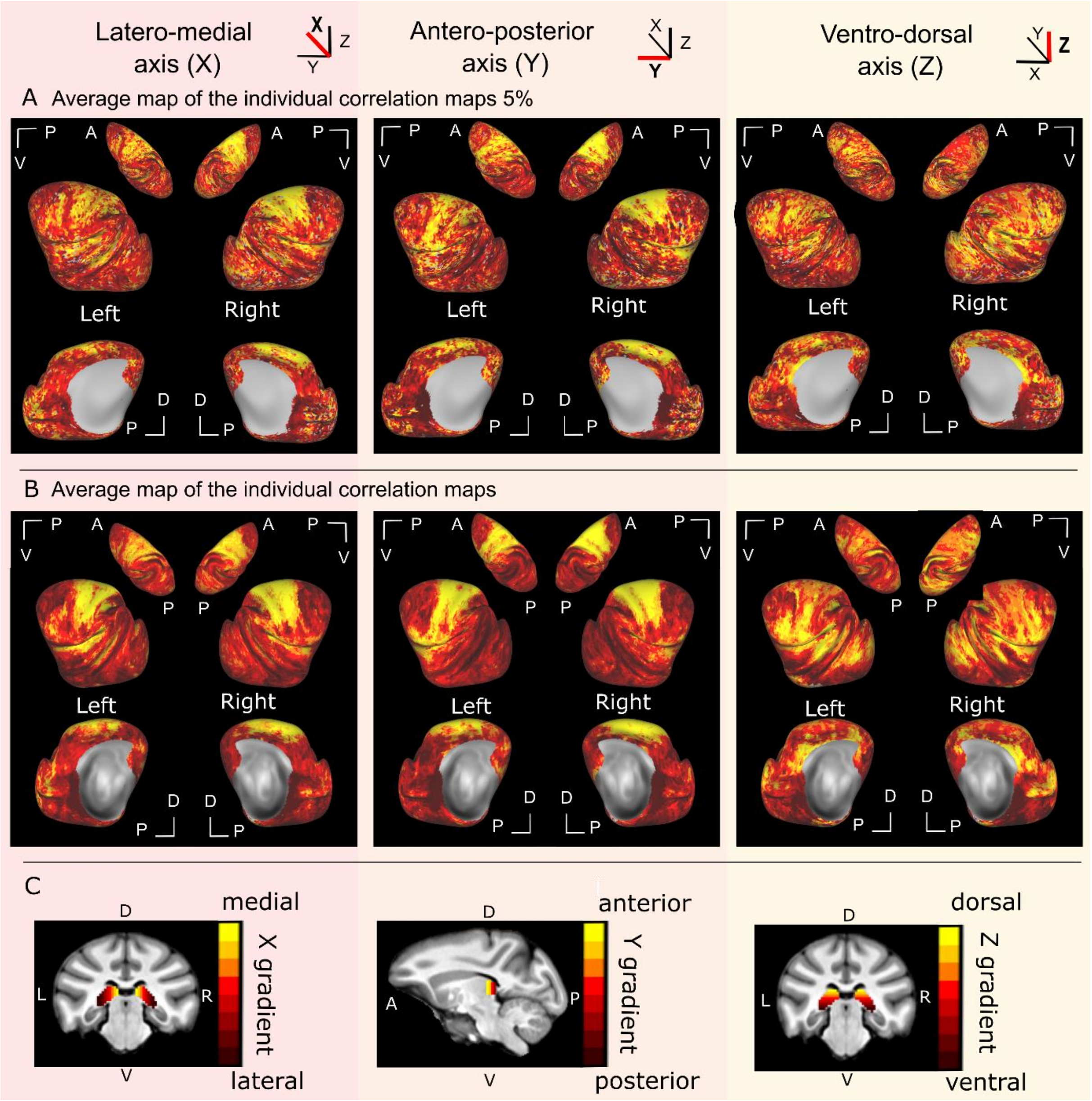

Figure S5: Global functional connectivity gradient of the pulvinar with the cortex. Comparison of 5% best winner-take all functional connectivity maps to the absolute best winner-take all functional connectivity maps. The pulvinar is subdivided into slices of 2mm thickness. These slices are used as seeds for a seed-to-whole brain functional connectivity analysis. A winner-take-all procedure is then applied to associate (color code) each cortical voxel with the pulvinar slice it maximally correlates with. The 5% winner-take-all associates each cortical voxel with the pulvinar slice it maximally correlates with and that shows 5% more correlation than the second best. Only ipsilateral correlations are presented. A) 5% Winner-take-all functional connectivity maps across the 10 monkeys. B) Mean winner-take-all functional connectivity maps across the 10 monkeys. C) Pulvinar slice seeds used in the functional connectivity analysis, for each of the analysis axes of interest. Details are provided in figure 1.

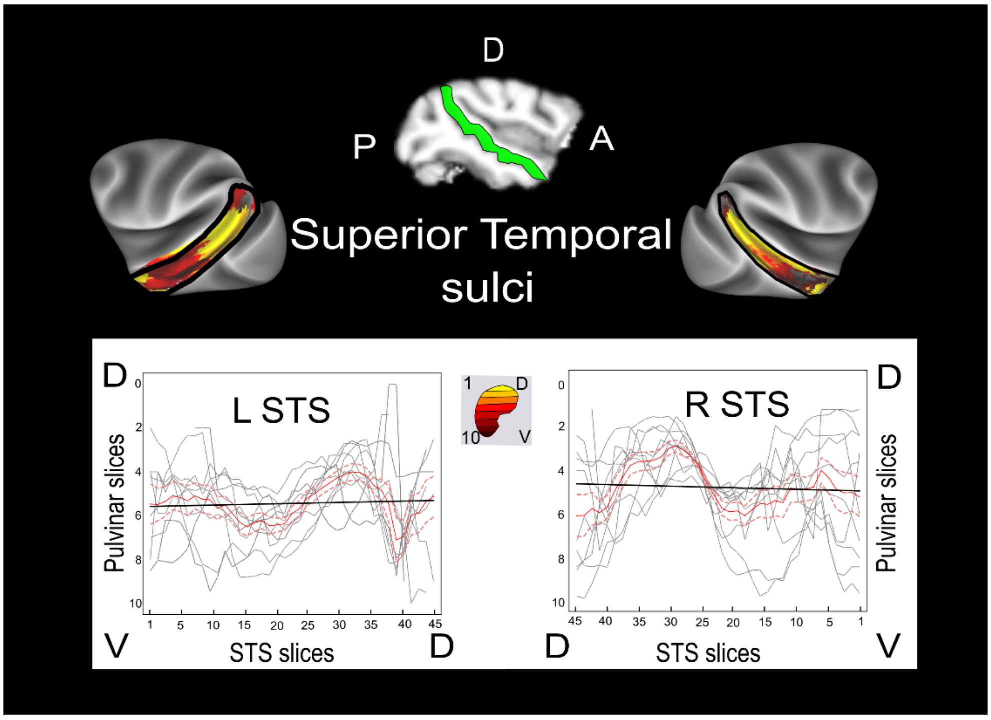

Figure S6: Local functional connectivity gradients of the pulvinar with the superior temporal sulcus. Only ipsilateral correlations between the STS and the pulvinar are presented for the STS along the ventro-dorsal axis. Individual subject (grey) and mean across all the subjects (red) correlations are presented as well as the group linear regression (black).

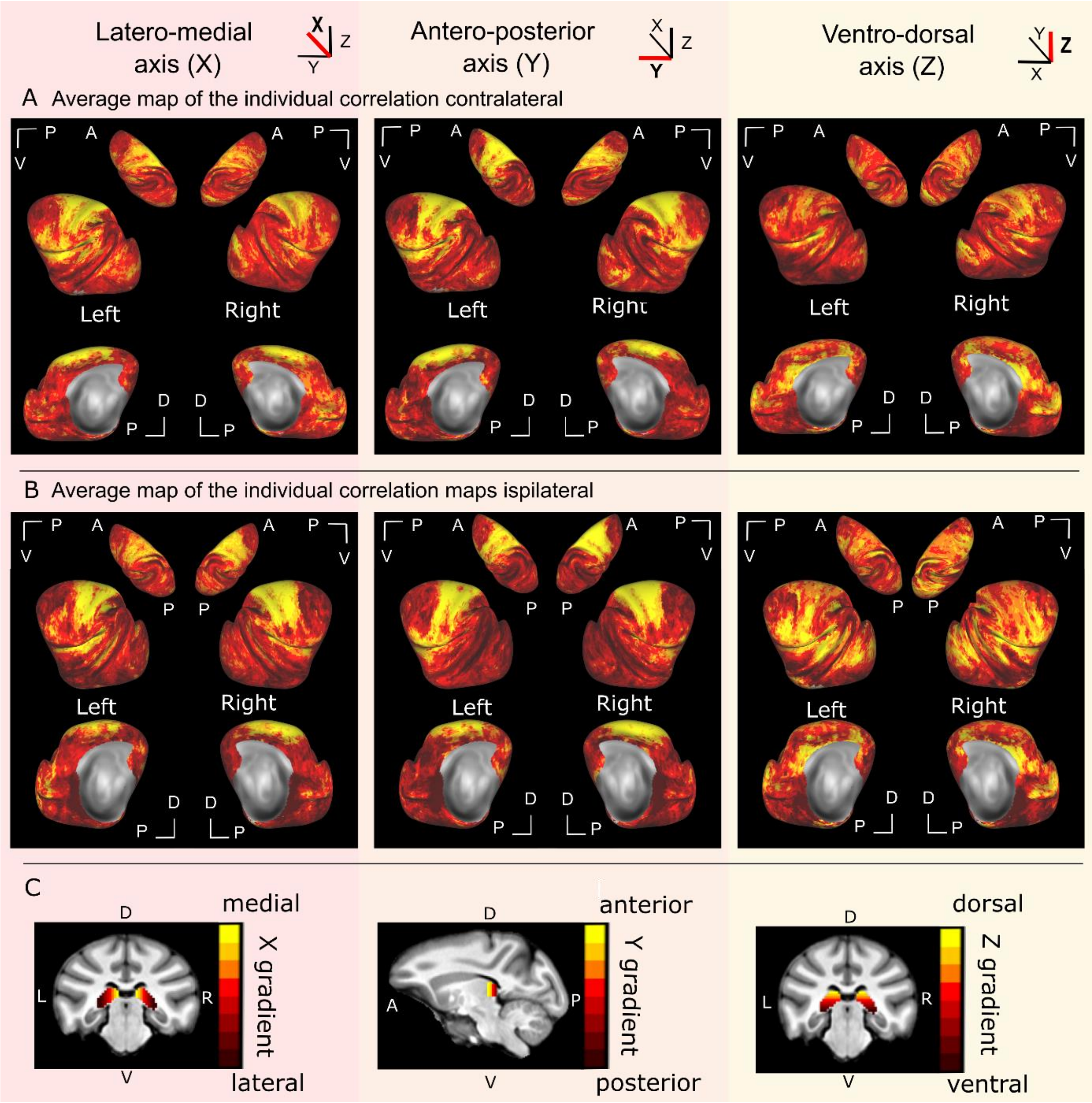

Figure S7: Global functional connectivity gradient of the pulvinar with the contralateral brain hemisphere. The pulvinar is subdivided into slices of 2mm thickness. These slices are used as seeds for a seed-to-whole brain functional connectivity analysis to the contralateral hemisphere. A winner-take-all procedure is then applied to associate (color code) each cortical voxel with the pulvinar slice it maximally correlates with. A) Winner-take-all functional contralateral connectivity maps across the 10 monkeys. B) Winner-take-all functional ipsilateral connectivity maps across the 10 monkeys Mean winner-take-all functional connectivity maps across the 10 monkeys. C) Pulvinar slice seeds used in the functional connectivity analysis, for each of the analysis axes of interest. Details are provided in figure 1.
